# Long-term outcomes of left atrial appendage closure techniques on stroke prevention of recurrent atrial fibrillation patients: epicardial excision versus percutaneous occlusion

**DOI:** 10.3389/fcvm.2025.1601303

**Published:** 2025-06-04

**Authors:** Adnan Abibe Goia, Meng Xu, Hu Qiuming

**Affiliations:** Department of Cardiovascular Surgery, Anzhen Hospital, Capital Medical University Affiliated Hospital, Beijing, China

**Keywords:** radiofrequency ablation, left atrial appendage, excision, occlusion, stroke

## Abstract

**Objective:**

This study aimed to compare the efficacy of left atrial appendage closure performed by excision (LAAC-EE) vs. occlusion (LAAC-PO) for stroke prevention in patients with recurrent atrial fibrillation undergoing radiofrequency ablation.

**Methods:**

In this retrospective analysis, 160 consecutive patients (109 undergoing LAAC-EE and 51 undergoing LAAC-PO) were evaluated. To adjust for baseline differences, stabilized inverse probability of treatment weighting (IPTW) was applied using a logistic regression model with age, sex, and CHA₂DS₂-VASc score as predictors. Weighted Kaplan–Meier survival analyses were conducted to assess stroke-free survival over a 5-year follow-up period, and weighted Cox proportional hazards regression was used to evaluate the association between LAAC modality and stroke occurrence, adjusting for age, sex, diabetes, CHA₂DS₂-VASc score, HAS-BLED score, and left atrium size.

**Results:**

Overall, the weighted mean CHA2DS2-VASc score was 3.1 ± 0.1 (3.0 ± 0.2 in LAAC-EE vs. 3.3 ± 0.2 in LAAC-PO; *p* = 0.159), indicating moderate baseline stroke risk. When stratified, 39.2% of LAAC-EE and 18.9% of LAAC-PO patients were in the low-risk category (CHA2DS2-VASc ≤2), 48.2% vs. 69.6% in the medium-risk group (score 3–4), and 12.6% vs. 11.5% in the high-risk group (score ≥5) (*p* = 0.093). Over 5 years, stroke occurred in 64 patients—29.4% in the LAAC-EE group vs. 62.7% in LAAC-PO—and weighted Kaplan–Meier analysis showed significantly greater stroke-free survival with excision (log-rank *p* < 0.001). In the weighted multivariate Cox model, LAAC-EE was associated with a non-significant 51.6% reduction in stroke risk (HR 0.48; 95% CI 0.13–1.74; *p* = 0.27). Age (HR 1.09 per year; *p* = 0.008) and HAS-BLED score (HR 10.54; *p* < 0.001) remained significant predictors, whereas sex, diabetes, and CHA₂DS₂-VASc score did not.

**Conclusion:**

Although the multivariate analysis did not achieve statistical significance for the treatment modality, the observed hazard ratio indicates that LAAC-EE may reduce stroke risk by approximately 51.6% compared to LAAC-PO. The significant impact of age and HAS-BLED score on stroke risk underscores the importance of individualized patient selection. These findings suggest a potential clinical benefit of LAAC-EE, particularly among lower-risk patients, and warrant further investigation in larger prospective studies.

## Introduction

Atrial Fibrillation (AF) is the most common arrhythmia affecting people on a worldwide scale. AF has a number of concerning complications such as: increased morbidity, recurrent hospitalizations, Heart Failure (HF) and Stroke. Stroke is specifically of high concern, as AF increases the risk of stroke 5-fold (1, 2). In the management of AF, rate and/or rhythm control techniques or medication are primarily used with the end-goal of preventing stroke and circulatory instability. Stroke risk in atrial fibrillation (AF) patients is traditionally stratified using the CHA₂DS₂-VASc score, with oral anticoagulation (OAC) previously recommended for men with scores ≥2 and women with scores ≥3. The HAS-BLED score has similarly been used to estimate bleeding risk and guide anticoagulation decisions. In our study, these tools were employed to assess baseline thromboembolic and bleeding risk, as they reflected standard clinical practice at the time of data collection. We acknowledge, however, that the 2024 ESC guidelines have since lowered the treatment threshold to CHA₂DS₂-VASc ≥1 for men and moved away from routine HAS-BLED scoring in favor of a more integrated bleeding risk assessment. Nonetheless, our analysis remains relevant to the clinical decision-making context during the study period (1, 3, 4).

More recently a procedure to potentially limit stroke altogether and limit/omit OAC in high bleeding risk patients with AF has been increasingly used, which consists of the surgical and percutaneous approaches to prophylactically exclude the left atrial appendage (LAA) (1). Recent data seems to suggest that AF-associated strokes lead to worse prognosis than those occurring in the absence of AF (5). Furthermore, there is substantial evidence that the left atrial appendage is an important source of thrombi in patients with AF and underlying heart disease. The majority of strokes in AF are associated with left atrial thrombi, found in approximately 15% of patients with non-valvular AF, with 90% located in the LAA (6–9).

The following retrospective study had at its premise, comparing 2 modalities of LAA Closure (LAAC), namely epicardial excision (EE) and percutaneous occlusion (PO) in order to determine its efficacy in stroke prevention when done concomitantly with radio-frequency ablation, based on the evaluation of the long-term postoperative outcomes.

## Methods

### Patient population

With the aforementioned goal in mind, we retrospectively analyzed a total population of 560 Recurrent AF patients who had undergone either one of the procedures in conjunction with radiofrequency ablation between 2015 and 2019 in our center. After applying inclusion and exclusion criteria a final sample of 160 patients was selected and followed them for a total of 60 months postoperatively. The institutional review board approved the research protocol, and all patients provided informed consent before surgery for possible data usage. Due to the retrospective nature of the study, requirement of informed consent for any other type of patient data was waived. The periprocedural patient data was collected from electronic medical records including clinical characteristics (AF type, CHA2DS2-VASc and HAS-BLED scores, etc.), peri- and postoperative events or complications and discharge medication.

Patients were selected for LAA closure based on a combination of thromboembolic risk, bleeding risk, and procedural feasibility. In both groups, all patients had recurrent AF (≥1 documented episode after initial ablation) and either contraindications to long-term oral anticoagulation (e.g., prior major bleeding, high HAS-BLED score) or a high CHA2DS2-VASc score indicating elevated ischemic risk. Inclusion criteria consisted of patients with recurrent AF—defined as at least one documented episode of AF following previous ablation—who underwent either excision or occlusion of the left atrial appendage concurrently with radiofrequency ablation. LAA excision was performed during thoracoscopic ablation and applied only to patients selected for surgical intervention. Percutaneous occlusion was reserved for patients who were unsuitable for or declined surgical options. We confirm that none of the patients undergoing LAA excision had attempted percutaneous occlusion prior. Exclusion criteria included a history of prior cardiac surgery, significant hepatic or renal dysfunction, heart failure, failed occluder implantation, incomplete clinical data, or inability to complete follow-up.

From the total cohort we selected 109 cases of surgical minimally invasive ablation plus left atrial appendage epicardial excision which became Group 1 (LAAC-EE), and 51 cases of catether ablation plus left atrial appendage percutaneous occlusion, Group 2 (LAAC-PO).

To control for potential confounders, we initially employed a propensity score-based approach. A logistic regression model was used to estimate the propensity scores, incorporating baseline characteristics such as age, CHA2DS2-VASc score, HAS-BLED score, hypertension, and diabetes. Nearest-neighbor 1:1 matching with a caliper of 0.2 was first attempted, which yielded 33 matched pairs; however, a substantial number of patients were excluded due to the absence of suitable matches. To preserve the full sample size and achieve better balance between the treatment groups, we subsequently applied stabilized inverse probability of treatment weighting (IPTW). Stabilized weights were derived from a logistic regression model that included age, sex, and CHA2DS2-VASc score as predictors of treatment assignment. All subsequent analyses, including weighted Kaplan–Meier survival curves and Cox proportional hazards regression, were performed on the IPTW-adjusted dataset.

We decided to compare them in terms of Stroke episodes, overall long-term postoperative outcomes and postoperative medical therapy. The primary outcome of interest was the development of stroke during follow-up.

### Procedure overview

Patients underwent left atrial appendage closure (LAAC) via two distinct approaches. Group 1 (LAAC-EE) received minimally invasive thoracoscopic surgical excision of the LAA combined with radiofrequency ablation. Group 2 (LAAC-PO) underwent percutaneous transcatheter occlusion using the Watchman (Boston Scintific, Natick, MA, USA) device during catheter ablation. All procedures were performed by experienced specialists under general anesthesia.

Detailed descriptions of the surgical and percutaneous techniques, including device deployment criteria, are provided in the Supplementary Materials (Supplementary Methods 1).

### Postoperative management

Antiarrhythmic drugs were continued after surgery for 3 months and then tapered off in the presence of a stable sinus rhythm (SR). Meanwhile, a b-blocker was served as rate-control medication according to postoperative heart rate.

Postoperative anticoagulation was in accordance with the instructions found in the American College of Cardiology/American Heart Association/European Society of Cardiology guidelines (10). Electrical cardioversion (ECV) was recommended if a patient had symptomatic AF lasting for more than 24 h.

### Follow-up

The patients were followed for a period of 60 months, with checkups starting at 1 year after the patients stopped the antiarrhythmic drugs (AAD), and every year subsequently. The first 3 months after operation was designed as blanking period. In the present study, as 95 of the patients (59.4%) lived outside of Beijing it was difficult for these patients to come to our center for each examination. We advised those who could not come to our center to undergo the ECG and echocardiographic examinations in their local city and to mail the results to us, and periodically checked-in with them via phone at the forementioned chekup times. If the patients developed a relapse or stroke of any kind during the follow-up period, we enquired and recorded the details of the recurrence.

### Statistical analysis

Continuous variables are presented as weighted means ± standard error (SE) or as medians with interquartile ranges, as appropriate. Categorical variables are reported as weighted effective numbers and percentages. Stabilized inverse probability of treatment weights (IPTW) were calculated using a logistic regression model with age, sex, and CHA2DS2-VASc score to adjust for baseline differences between treatment groups.

Between-group comparisons for continuous variables were performed using weighted Student's *t*-tests (or the Mann–Whitney *U*-test if the data were non-normally distributed), while differences in categorical variables were assessed using weighted chi-square tests or Fisher's exact test, as appropriate. Kaplan–Meier survival curves were constructed using the weighted data and compared via the log-rank test. Weighted Cox proportional hazards regression models were used to evaluate the association between LAAC procedure type and the occurrence of stroke, with hazard ratios (HRs) and their 95% confidence intervals (CIs) reported.

All analyses were conducted using R (version 4.4.1) and IBM SPSS Statistics (version 22.0 IBM Corp., Armonk, NY, USA). A two-sided *p*-value of <0.05 was considered statistically significant.

## Results

### Baseline characteristics

A total of 160 consecutive patients underwent LAAC in our center, 109 EE and 51 PO. Using stabilized inverse probability of treatment weighting (IPTW) to adjust for baseline differences, the effective sample sizes were balanced between the two modalities, with the LAAC-PO and LAAC-EE groups showing comparable distributions on most baseline parameters.

With regards to the preoperative diagnosis of AF type in the two groups, the weighted distribution differed between the groups. In the LAAC-PO group, the effective counts and weighted proportions were as follows: Paroxysmal AF, 8.1 (14.4%); Persistent AF, 22.6 (40.4%); and Long-standing AF, 25.2 (45.1%). In contrast, the LAAC-EE group had 29.0 (28.1%) Paroxysmal, 32.5 (31.5%) Persistent, and 41.8 (40.4%) Long-standing AF cases. Although these differences suggest a trend toward a higher prevalence of Paroxysmal AF in LAAC-EE and of Persistent/Long-standing AF in LAAC-PO, the overall difference in AF type distribution was not statistically significant (*p* = 0.371).

Overall weighted mean CHA2DS2-VASc score was 3.1 ± 0.1, with the male participants presenting a higher number of scores 1, whilst in the female participants a higher number of patients with scores of 4 were found. Further analysis of the weighted mean CHA2DS2-VASc score was determined to be 3.0 ± 0.2 in LAAC-EE and 3.3 ± 0.2 in LAAC-PO (*p* = 0.159), indicating a moderate risk of stroke in both groups. Scoring for the two patient groups was done while focusing on both overall scores and the distribution of patients within three subcategories: low for those with scores less than or equal to 2, medium for those with scores equal to 3 or 4, and high for those with scores of 5 or higher. This subsequent weighted stratification based on a CHA2DS2-VASc score threshold into different subcategories—low, medium, and high—revealed that 40.5 (39.2%) of individuals in the LAAC-EE group and 10.5 (18.9%) in the LAAC-PO group fell into the low-risk category. The medium-risk category included 49.8 (48.2%) in the LAAC-EE group and 38.9 (69.6%) in the LAAC-PO group. In contrast, the high-risk category showed 13.0 (12.6%) in the LAAC-EE group and 6.4 (11.5%) in the LAAC-PO group. The observed differences in the distribution of scores between the groups were statistically insignificant (*p* = 0.093).

Additionally, the mean HAS-BLED score was 1.4 ± 0.1 in LAAC-EE and 1.6 ± 0.1 in LAAC-PO, with a statistically insignificant difference (*p* = 0.232).

Patients' age ranged from 30 to 88 years (M = 63.2, SD = 9.9). In terms of Body Mass Index both groups showed similar numbers, group 1 with 25,3% and group 2 with 25,4%. Patients characteristics are detailed in Table 1.

**Table 1:** Baseline characteristics.

Baseline characteristics	Group 1-LAAC-EE	Group 2-LAAC-PO	*P*-value
Age (years)			
Mean	62.4 ± 1.0	64.4 ± 1.3	0.235
Sex			
Male *n* (%)	66 (64.1%)	29 (52.5%)	0.380
Female *n* (%)	37 (35.9%)	27 (47.5%)	0.380
BMI (body mass index) (%)	25.3 ± 0.2	25.4 ± 0.5	0.910
Type of AF *n* (%)			
Paroxysmal	29.0 (28.1%)	8.1 (14.4%)	0.371
Persistent	32.5 (31.5%)	22.6 (40.4%)	
Long-standing	41.8 (40.4%)	25.2 (45.2%)	
Medical history *n* (%)			
Diabetes	18.8 (18.2%)	9.6 (17.1%)	0.883
Hyperthyroidism	0.8 (0.7%)	4.0 (7.2%)	0.022
Hypertension	61.4 (59.5%)	26.0 (46.5%)	0.296
CHA2DS2-VASc score	3.0 ± 0.2	3.3 ± 0.2	0.159
CHA2DS2-VASc score low (0–2)	40.5 (39.2%)	10.5 (18.9%)	0.093
CHA2DS2-VASc score medium (3–4)	49.8 (48.2%)	38.9 (69.6%)	
CHA2DS2-VASc score high (≥5)	13.0 (12.6%)	6.4 (11.5%)	
HAS-BLED score	1.4 ± 0.1	1.6 ± 0.1	0.232
Pre-operative ejection fraction (%)	62.4 ± 0.7	61.9 ± 1.8	0.787
Pre-operative left atrium size (mm)	41.4 ± 0.4	39.9 ± 0.4	0.006
Pre-operative left ventricular end diastolic diameter (mm)	47.7 ± 0.6	48.9 ± 0.7	0.160

Data are presented as weighted effective numbers (*n*, which may be fractional) and weighted percentages (%) or weighted means ± standard error (SE), derived using stabilized inverse probability of treatment weighting (IPTW). Note that the effective numbers represent the adjusted sample sizes after weighting and may not match the raw counts exactly.

The total number of patients from both groups undergoing pharmacological therapy included: 31 (19.4%) on novel oral anticoagulants (NOACs; e.g., Dabigatran or Rivaroxaban), 31 (19.4%) on warfarin, 55 (33.1%) on antiplatelet therapy (aspirin), 5 (3.1%) on clopidogrel, 69 (43.1%) on beta-blockers, 8 (5.0%) on amiodarone, 2 (1.3%) on digoxin, and 1 (0.6%) with pacemaker implantation. Pharmacological Therapy intake differences can be seen detailed in the Supplementary Table S3.

### Clinical outcomes

A total of 16 (10%) patients passed away during the duration of the study, 7 (6.4%) from the LAAC-EE group and 9 (17.6%) from LAAC-PO group. Deaths from unknown reasons, where family members did not know the cause of death or did not want to speak about it, were observed in 7 of the 16 patients (43.8%).

Death from cerebral infarction was observed in 3 patients (18,8%) and 3 patients died from lung cancer (18,8%). 1 patient passed away due to having contracted COVID-19 (6.3%), another 1 patient passed away from bile duct cancer (6.3%) and another 1 from breast cancer (6.3%). See Table 2 for more details.

**Table 2:** Post-operative outcomes for both modalities of LAAC.

Post operative outcomes	Group 1-LAAC-EE	Group 2-LAAC-PO	*P*-value
Post operative ejection fraction (%)	62.9 ± 6.1	65.5 ± 4.1	0.198
Post operative left atrium size (mm)	39.4 ± 4.0	40.7 ± 4.8	0.273
Post operative left ventricular end diastolic diameter (mm)	45.8 ± 5.7	48.2 ± 5.3	0.041

Data are presented as weighted means ± standard error (SE) or weighted effective numbers (%), calculated using stabilized IPTW. These values reflect the adjusted estimates that account for baseline differences between the groups.

After post procedural ECG, a total of 88 patients (55%) demonstrated sinus rhythm, 53 patients (33%) showed a relapse to AF, and 13 patients (8.1%) did not have any recorded ECG. There was also a case of 4 patients (2.5%) in which it was registered Atrial Flutter, as well as the case of 1 patient (0.6%) with sinus bradycardia and 2 others (1.2%) in which a pacemaker implant was performed. 1 patient (0.6%) had to undergo defibrillation twice due to AF episodes.

There's registry of 13 patients (8.1%) which had episodes of postoperative cerebral infarction TIA. 2 patients suffered cerebral hemorrhages (1.2%) and 1 patient (0.6%) suffered from limb embolism. Group Modality can be seen detailed in the Supplementary Table S4. Throughout the follow-up period, all patients demonstrated recurrence of AF at some point in time.

### Follow-up outcome and Cox proportional hazard regression adjusting

**Figure 1:**
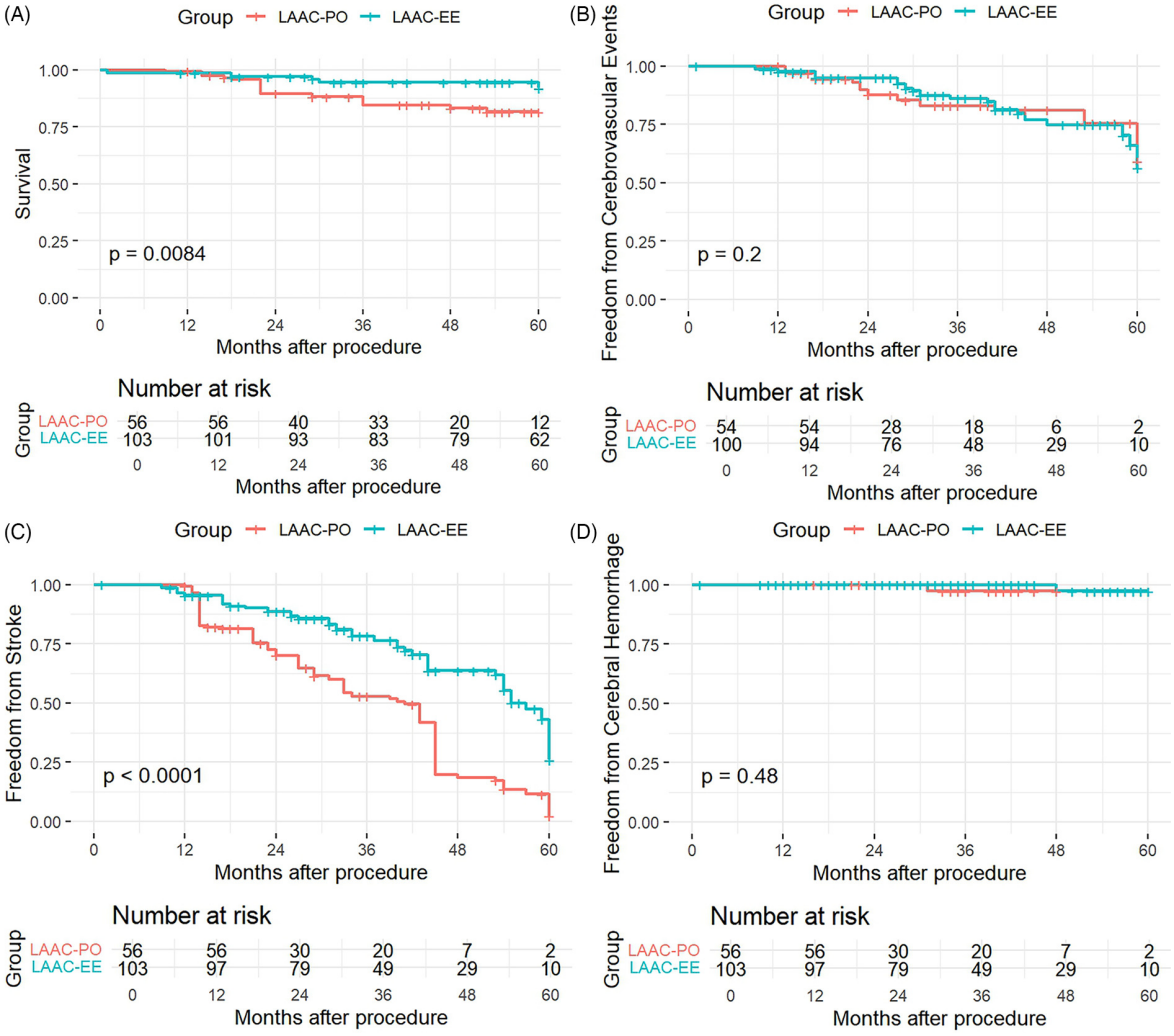
Kaplan–Meier curve of survival **(A)**, freedom from cerebrovascular events **(B)** stroke ocurrences **(C)** and cerebral hemorrhage **(D)** for both modalities of LAAC.

After 5 years of follow-up, stroke occurred in 64 patients, from which 32 (29.4%) were in the excision group and 32 (62.7%) in the occlusion group. The weighted K-M analysis showed that patients in the occlusion group were associated with a higher risk of stroke than those in the excision group (Log-rank *P* < 0.001) over a period of 60 months post-procedure, as demonstrated in Figure 1.

A Cox proportional hazards regression model was performed using stabilizing IPTW weights, which adjusted for age, sex, diabetes, CHA2DS2-VASc score, HAS-BLED score, to adjust the association between procedure types and the occurrence of stroke. After adjusting for these covariates, the analysis showed that for treatment effect (LAAC-EE vs. LAAC-PO) the hazard ratio (HR) for LAAC-EE compared to LAAC-PO was 0.484 (95% CI: 0.1349–1.738, *p* = 0.266), suggesting a 51.6% reduction in the hazard of stroke for LAAC-EE. However, this difference was not statistically significant. Age on the other hand was a significant predictor of stroke (HR = 1.0906, 95% CI: 1.018–1.175, *p* = 0.008), with each additional year of age increasing the hazard of stroke by 9.1%. The HAS-BLED score was also a significant predictor (HR = 10.5399, 95% CI: 4.2530–26.1206, *p* < 0.001), highlighting its importance in assessing stroke risk. The other variables Sex, CHA2DS2-VASc did not show statistically significant effects in this model (*p* > 0.05).

The Kaplan–Meier survival curves compared outcomes between two patient groups, LAAC-EE and LAAC-PO, as follows:

The K-M curve for overall Survival (Panel A) revealed a statistically significant difference between the LAAC-EE and LAAC-PO groups (*p* = 0.0084). The survival probability was consistently higher for the LAAC-EE group throughout the follow-up period. Particularly after 24 months, the survival curve for the LAAC-PO group showed a more pronounced decline, indicating that patients in the LAAC-EE group had better survival outcomes compared to those in the LAAC-PO group.

The analysis of freedom from cerebrovascular events graph (Panel B) demonstrated a insignificant statistical difference between the two groups, with a *p*-value of 0.2, with the survival curves of the LAAC-EE group and the LAAC-PO group being similar.

The KM curve for freedom from stroke (Panel C) showed a very significant difference between the groups, with a *p*-value of less than 0.0001. Patients in the LAAC-EE group had a substantially higher likelihood of remaining stroke-free over time compared to the LAAC-PO group. The separation between the curves was evident early in the follow-up period and continued to widen, indicating a pronounced protective effect against stroke for the LAAC-EE group.

Panel D presents the analysis for freedom from cerebral hemorrhage, which reveals no statistically significant difference between the two groups (*p* = 0.48). Both groups have similar probabilities of remaining free from cerebral hemorrhage throughout the 60-month follow-up period, as reflected in the overlapping confidence intervals and the non-significant *p*-value. This suggests that the type of procedure does not influence the rate of cerebral hemorrhage.

**Figure 2:**
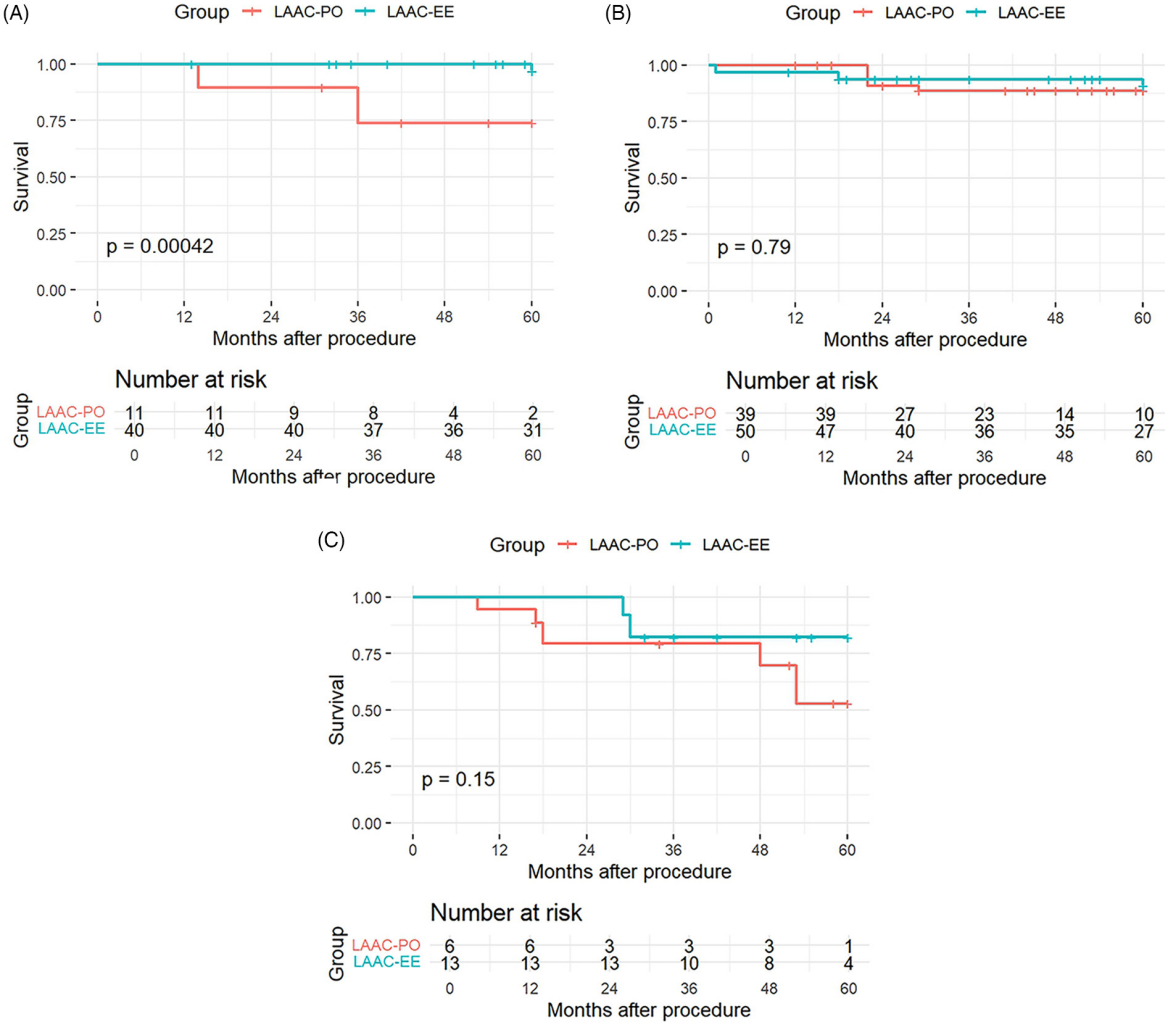
Kaplan–Meier curves for CHA2DS2-VASc risk subroups: low-risk subgroup **(A)**, medium-risk subgroup **(B)**, high-risk subgroup **(C)**.

Stratified K–M graphs were ploted for the 3 CHA2DS2-VASc subroups and in the low-risk subgroup (0–2) there was a statistically significant difference between treatment groups (*p* = 0.00042), suggesting that among patients with low CHA2DS2-VASc scores, survival differed between LAAC-EE and LAAC-PO. In stark contrast, for the medium-risk subgroup (3–4) and in the high-risk subgroup (≥5), no significant difference was detected in either subgroup (*p* = 0.79 and 0.15 respectively), as the medium-risk subgroup survival curves between the two treatments were very similar and despite there being a trend in the high-risk subgroup. The K-M plots for the subgroups are shown in Figure 2.

The number of patients at risk at various time points during follow up (0, 12, 24, 36, 48, and 60 months) is provided for each panel. This information is crucial as it shows the decreasing number of patients over time due to events or censoring, providing context for the survival probabilities presented in the Kaplan–Meier curves.

These findings suggest that the LAAC-EE procedure may offer a protective benefit in reducing mortality and stroke risk, but it does not appear to significantly alter the overall rate of cerebrovascular events (which we define to include transient ischemic attacks and other nonfatal ischemic or hemorrhagic brain injuries) or the incidence of intracranial hemorrhage. Furthermore this effect may be most evident to patients with low CHA2DS2-VASc scores, whereas in medium and high-risk groups, the survival differences are negligible.

**Figure 3:**
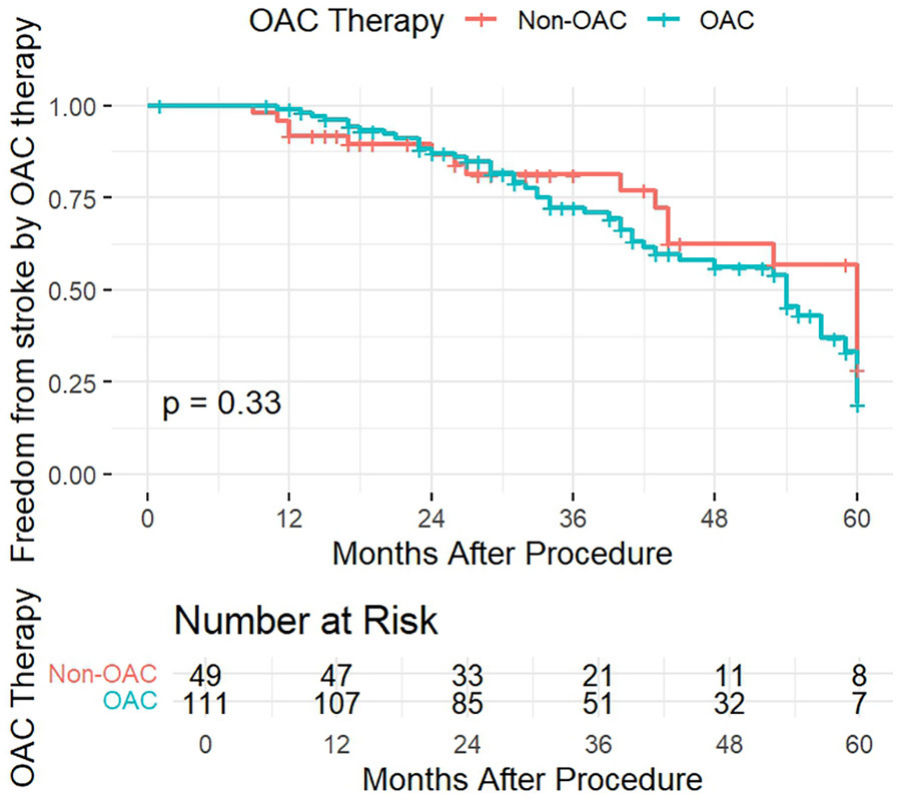
Kaplan–Meier curve of freedom from stroke according to OAC therapy.

To verify the effect of OAC therapy on freedom from stroke we also divided the patients into therapy groups, between those who underwent OAC therapy and those who did not (Non-OAC). The KM curve for freedom from stroke according to OAC therapy (Figure 3) displayed an insignificant difference between the groups, with a *p*-value of 0.33. Patients in both groups had a similar likelihood of remaining stroke-free over time, with the curves diverging slightly in the follow-up period, yet the diference was statistically insignificant.

## Discussion

The main goal of this study was to assess the efficacy of two different modalities of LAAC, namely EE and PO, for the prevention of stroke in patients with recurrent AF undergoing radiofrequency ablation by evaluating long-term outcomes.

In recent years, hybrid One-stop techniques combining radiofrequency ablation with LAAC have demonstrated efficacy in preventing cardioembolic events in patients who poorly tolerate OAC therapy (1). Studies suggest that radiofrequency ablation maintains sinus rhythm more effectively than drug therapy, improves cardiac function, and reduces readmission rates and mortality (11, 12). LAAC, in turn, has been shown to prevent thromboembolic events and bleeding complications, demonstrating non-inferiority to NOACs (13).

In our study, stroke occurrence was significantly higher in the occlusion group (62.7%) compared to the excision group (29.4%). Mortality was also higher in the occlusion group (17.6%) compared to the excision group (6.4%) (26). These findings suggest that LAAC-EE may be more effective in preventing stroke, though this effect was primarily observed in patients with a low CHA2DS2-VASc score.

A Cox proportional hazards model using stabilized IPTW weights found that the hazard ratio for LAAC-EE compared to LAAC-PO was 0.48 (95% CI: 0.13–1.74, *p* = 0.27) after adjusting for age, sex, and CHA2DS2-VASc score. Although the point estimate suggests a potential survival benefit for LAAC-EE, the effect was not statistically significant, likely due to the limited number of events (16 deaths among 160 patients). These findings indicate that while there is a trend toward improved survival with LAAC-EE, the study is underpowered to definitively demonstrate a treatment benefit.

Although our study suggests that LAAC-EE may be a more effective and favorable strategy than LAAC-PO, prior studies have highlighted that LAAC-PO remains a promising strategy for perioperative stroke reduction, particularly for patients with contraindications to OAC (13–15). The growing interest in LAAC-PO, along with concerns regarding surgical excision techniques, has led to advancements in newer surgical devices designed primarily for occlusion, such as the Watchman and Amplatzer devices (13, 16, 17, 27).

Guarracini et al. (18) recently reviewed the feasibility of both LAAC-EE and LAAC-PO for stroke prevention, emphasizing the need for individualized treatment selection. Furthermore, studies have demonstrated the cost-effectiveness of LAAC procedures for stroke prevention in patients with AF and increased stroke risk, particularly those who cannot tolerate OAC (19, 20).

Interestingly, despite a higher proportion of patients with medium CHA2DS2-VASc scores in the LAAC-PO group, a relatively higher number of stroke episodes and deaths were observed in this group. Prior research suggests that CHA2DS2-VASc is a reliable predictor of AF recurrence (21, 22). The increased stroke risk in the LAAC-PO group may stem from various factors, including potential peridevice leakage or residual shunting (23). The pathophysiological rationale behind differences in stroke risk between LAAC-EE and LAAC-PO lies in the completeness and permanence of LAA exclusion. LAAC-EE removes or entirely isolates the LAA from the circulation, leaving minimal to no residual stump and thereby markedly reducing sites for thrombus formation when performed correctly (6). By contrast, LAAC-PO relies on an endocardial device [e.g., Watchman (Boston Scintific, Natick, MA, USA)] to seal off the appendage, but even with optimal sizing and positioning, it may leave small residual leaks or incomplete coverage that can serve as niduses for thrombus (23). Furthermore, the process of endothelialization over the device surface varies among patients; delayed or uneven tissue ingrowth can expose pro-thrombotic surfaces during the early post-implantation period (8). These anatomical and healing-related differences likely underlie the lower incidence of ischemic events observed in our LAAC-EE cohort, a finding mirrored in prior registry data (6, 8, 23).

In a retrospective analysis, Hernandez et al. (24) advocated for LAAC-EE as a viable option in cases where PO may lead to poor outcomes, arguing that “in an era of expanding percutaneous and medical treatment options, it is important to recall the utility of therapies that may seem novel in contemporary practice but had been previously used with success and remain viable options”. Our results support this perspective, as both procedures demonstrated efficacy in LAA closure.

Recent studies suggest that, in the short term, LAAC-PO may lead to positive remodeling of the LAA due to device occlusion, resulting in loss of compression and potential peridevice leak (23). However, the clinical significance of these leaks remains unclear, and no such findings were observed in our study during follow-up.

A more patient-centric approach may be warranted, potentially leading to the establishment of heart-team treatment strategies based on patient characteristics to optimize LAAC selection (25). Branzoli et al. highlighted the importance of a tailored approach in determining the most appropriate LAAC strategy for individual patients (25).

Although the adjusted analysis did not reach statistical significance (*p* = 0.27), the hazard ratio of 0.48—indicating a 51.6% reduction in stroke risk with LAAC-EE compared to LAAC-PO—remains clinically important and merits further investigation in larger, prospective studies.

This study has several limitations. Firstly, as a retrospective study, it is subject to biases from incomplete information, data limitations, and follow-up issues, potentially leading to inaccuracies. Some patients could not be reached or declined further follow-up. In particular, reliance on telephone follow-up for some patients may have introduced response bias or recall bias, which could affect data quality. Nonetheless, the available data provided a robust foundation for analysis. Secondly, follow-up methodologies may have led to underestimation of deaths, though the robustness of the dataset mitigates this concern. Thirdly, the relatively small sample size limits statistical power; however, the findings still provide valuable insights for future studies. Lastly, the COVID-19 pandemic introduced additional challenges in patient follow-up, which may have introduced minor biases in response rates.

Further research with larger cohorts, prospective trials and extended follow-up periods is necessary to confirm the findings of this study and refine treatment recommendations for LAAC procedures.

## Conclusion

Atrial fibrillation remains the most common cardiac arrhythmia worldwide, significantly increasing the risk of stroke and other thromboembolic events. Research into different left atrial appendage closure (LAAC) strategies is essential for optimizing stroke prevention, reducing mortality, and improving patient outcomes.

In this study, LAAC-EE demonstrated a relative advantage over LAAC-PO in reducing stroke occurrence, maintaining sinus rhythm, and achieving lower overall mortality. The survival benefit was most pronounced in patients with lower CHA2DS2-VASc scores, suggesting that baseline stroke risk may influence the effectiveness of LAAC strategies. While the weighted Cox proportional hazards analysis indicated a trend toward improved survival with LAAC-EE, statistical significance was not reached, likely due to the limited number of events.

From a clinical perspective, these findings support a more individualized approach to LAAC, where procedural selection is tailored based on patient-specific stroke risk, bleeding risk, and anatomical considerations. The role of LAAC-EE as a potentially superior strategy in select patient populations warrants further investigation. Additionally, the long-term outcomes observed in this study emphasize the importance of structured post-procedure follow-up and anticoagulation management to maximize the benefits of LAAC.

Future prospective studies with larger sample sizes and extended follow-up periods are needed to validate these findings and refine patient selection criteria. In the evolving landscape of AF management, a multidisciplinary “heart team” approach may help guide procedural decisions and improve long-term patient outcomes.

## Data Availability

The original contributions presented in the study are included in the article/Supplementary Material, further inquiries can be directed to the corresponding author.
