# Supplementary material for: Long-term outcomes of left atrial appendage closure techniques on stroke prevention of recurrent atrial fibrillation patients: epicardial excision versus percutaneous occlusion
Source: Front Cardiovasc Med. 2025 Jun 4;12:1601303. doi: 10.3389/fcvm.2025.1601303 (PMC12174162; doi:10.3389/fcvm.2025.1601303)
Supplement: Supplementary file 1 [file Presentation1.pdf]

Supplementary data

Supplementary Methods 1: Procedural Details

The procedure done to exclude the LAA was different in both groups 1 and 2:

1.1 LAAC with Epicardial Excision (LAAC-EE)

For the patients of group 1, a minimally invasive surgical procedure was performed by a team of experienced surgeons and it followed as such: The procedure was conducted after achievement of general anesthesia administered with a double-lumen endotracheal tube. Transesophageal echocardiography was performed in the operating room to verify the absence of a left atrial thrombus before the start of the procedure and adequacy of LAA excision at the end of the procedure. Surgical Wolf Mini-maze procedure was used and it involved making 3 small incisions in the patient's bilateral intercostal space, under the monitoring of the thoracoscopic field of vision, using a bipolar radiofrequency device (Atricure TM) for ablation treatment. Its main operations included: extensive isolation of bilateral pulmonary veins; linear ablation of the left atrium; partial denervation of the epicardium; and resection of the LAA.

The patient was first positioned with his left side down and the right arm abducted above his head in order to first ablate the right pulmonary veins. 3 incisions were made for better access and full direct view of the right side of the heart and pulmonary veins with ablation of both the right pulmonary veins and linear ablation of the left atrium performed with 2 or more overlapping lesions being created to ensure isolation. Ablation was deemed successful with ablation lines being visible and the conductance of the tissue decreased to less than 0.0025 siemens, indicating that the lesions were transmural. The patient was

repositioned with the right side down and the left arm above the head and the technique was repeated on the left side with the addition of division of the ligament of Marshall.

After ablation, the LAA excision was performed, verified on transesophageal echocardiography. The pericardium is closed on the left side. If the patients are not in sinus rhythm by the end of the procedure, they are positioned supine and given a synchronized direct-current shock to establish sinus rhythm. Extubation was routinely performed in the operating room.

1.2 LAAC with Percutaneous Occlusion (LAAC-PO)

In group 2, the procedure was done by a team of experienced interventional cardiologists and comprised transcatheter radiofrequency ablation and LAA Closure both of which are done under anesthesia. The catheter was inserted through the femoral vein, and a complete atrial septal puncture was performed under X-ray guidance. The puncture point was then adjusted according to the relative height of the LAA, to objectively make the tube sheath and the LAA axially consistent after puncture. Circular pulmonary vein isolation was performed, and either linear ablation and/or matrix modification were performed according to the surgeon's choice. If AF was still observed after ablation, intracardiac synchronous electrical cardioversion (10~20 J) was performed. After ablation, the LAA occlusion was performed with an occluder. The hardened guide wire was exchanged, the occluder guide system was replaced, and the pigtail catheter was delivered through the sheath. When the left atrial pressure was determined to be $\geqslant$10 mmHg (1 mmHg=0.133 kPa), LAA angiography was performed. The model and size of Watchman (Boston Scintific, Natick, MA, USA) occluder used was determined by the shape and opening diameter of the left atrial appendage. The PASS principle (position-anchor-size-sealing principle) had to be met before the LAA

occluder could be released. After occlusion of the LAA with an occluder and according to the surgeon's choice, either an esophageal ultrasound examination was performed immediately to evaluate the occlusion, or the residual shunt was evaluated according to fluoroscopy imaging. The process would be deemed a satisfactory occlusion if the position of the occluder was good and stable, the auricle orifice was completely covered, and the residual shunt on the edge of the occluder was less than 5mm.

Pharmacological Therapy	Group 1-LAAC-EE	Group 2-LAAC-PO
Aspirin	38(34.9%)	15(29.4%)
NOAC's	13(11.9%)	18(35.3%)
Warfarin	28(25.7%)	3(5.9%)

Table 3. Pharmacological therapies admnistered per surgical modality during post-operative and follow-up periods. *Data is presented as n (%)*

Stroke Type	Group 1-LAAC-EE	Group 2-LAAC-PO
TIA	6(3.8%)	7(4.4%)
Cerebral Hemorrhaege	1(0.6%)	1(0.6%)
Limb Embolism	0	1(0.6%)

Table 4. Stroke Type per surgical modality during follow-up period. *Data is presented as n (%)*

Figure Legends

Figure 1. Kaplan-Meier curve of Survival (A), freedom from Cerebrovascular events (B) Stroke ocurrences (C) and Cerebral Hemorrhage (D) for both modalities of LAAC.

Figure 2.Kaplan-Meier curves for CHA2DS2-VASc risk subroups: low-risk subgroup (A), medium-risk subgroup (B), high-risk subgroup (C)

Figure 3. Kaplan-Meier curve of freedom from stroke according to OAC therapy.
